# Laboratory Rodent Diets Contain Toxic Levels of Environmental Contaminants: Implications for Regulatory Tests

**DOI:** 10.1371/journal.pone.0128429

**Published:** 2015-07-02

**Authors:** Robin Mesnage, Nicolas Defarge, Louis-Marie Rocque, Joël Spiroux de Vendômois, Gilles-Eric Séralini

**Affiliations:** 1 University of Caen, Institute of Biology, EA2608 and Network on Risks, Quality and Sustainable Environment MRSH, Esplanade de la Paix, 14032 Caen Cedex, France; 2 CRIIGEN, 40 rue Monceau, 75008, Paris, France; Carleton University, CANADA

## Abstract

The quality of diets in rodent feeding trials is crucial. We describe the contamination with environmental pollutants of 13 laboratory rodent diets from 5 continents. Measurements were performed using accredited methodologies. All diets were contaminated with pesticides (1-6 out of 262 measured), heavy metals (2-3 out of 4, mostly lead and cadmium), PCDD/Fs (1-13 out of 17) and PCBs (5-15 out of 18). Out of 22 GMOs tested for, Roundup-tolerant GMOs were the most frequently detected, constituting up to 48% of the diet. The main pesticide detected was Roundup, with residues of glyphosate and AMPA in 9 of the 13 diets, up to 370 ppb. The levels correlated with the amount of Roundup-tolerant GMOs. Toxic effects of these pollutants on liver, neurodevelopment, and reproduction are documented. The sum of the hazard quotients of the pollutants in the diets (an estimator of risk with a threshold of 1) varied from 15.8 to 40.5. Thus the chronic consumption of these diets can be considered at risk. Efforts toward safer diets will improve the reliability of toxicity tests in biomedical research and regulatory toxicology.

## Introduction

Rodent feeding trials are the most widely used experiments in biomedical research and are particularly used to study the potential side effects of commercial products in mammals. They do not only constitute a test for human health but also for the environment. The rat may also be considered as a toxicological model for small mammals, either wild or kept as farm animals or pets. The quality of the rodent diet is thus crucial. Rodent diets are mostly formulated with agricultural products and by-products, and are susceptible to contamination with toxic environmental contaminants [1]. However, the extent and worldwide variability of this contamination has never been described. We have thus measured residues of 262 pesticides, 22 genetically modified organisms (GMOs), 4 heavy metals, 18 polychlorinated biphenyls (PCBs) and 17 polychlorinated dibenzo-p-dioxins and dibenzofurans (PCDD/Fs) in 13 rodent diets. These samples derived from 13 suppliers from 9 countries on 5 continents (North and South America, Europe, Asia, Africa and Oceania), representative of diets used in academic research and regulatory assessment.

These contaminations could participate to explain why populations of laboratory rodents across the world develop high rates of so-called “spontaneous” diseases. For instance in Sprague-Dawley rats from Harlan after 2 years, the mean incidences of mammary fibroadenomas and pituitary adenomas among control populations were 71 and 42% respectively [2]. The same strain from Charles River had means of 38% (13 to 62%) mammary fibroadenomas and 71% (26 to 93%) pituitary adenomas [3]. Moreover, these incidences were not stable, but increased or diminished over time [4]. It indicates that differences among rat populations cannot only be explained by genetic drift and may arise from different environmental conditions, including feed or water contamination. This work was conducted to test the extent of the feed contamination from 5 continents, and to deduce chemical exposures and hazards from regulatory official calculations (EPA guidelines). In fact, it is known that the mortality of laboratory rats has an extremely unexplained wide range, from 38 to 83% after 2 years [3], it is in general less for some Wistar rat strains [5].

These statistics are used as external controls for regulatory chronic tests. Treated rats are not only compared with the internal control of the experiment, but are subsequently compared with this whole population, represented by the compilation of all control groups formed by the past experiments of the laboratory, or on the rat strain, called “historical control data”. Historical controls are assumed to be of importance in the interpretation of regulatory chronic tests, and they are thus used to determine the biological significance of a statistical difference between the experimental animals and the concurrent controls. This does not usually apply to academic research, in which treated groups are only compared to concurrent matched controls, raised in the same conditions, fed with the same diet, except for one studied parameter.

**Fig 1:**
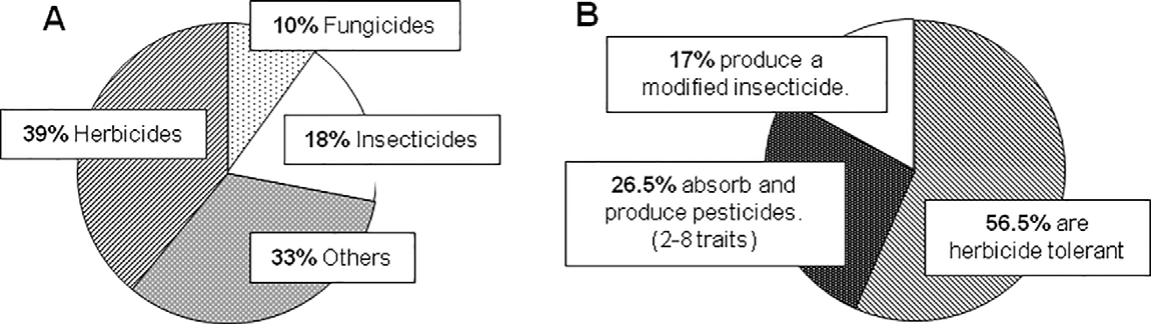
Pesticides and agricultural GMOs used worldwide. Data from ‘Pesticides Industry Sales and Usages Report: 2006 and 2007 Market Estimates’ [13] and ‘Global Status of Commercialized Biotech/GM Crops: 2013 [14]. (A) Data for pesticides represent 2006 and 2007 EPA estimates based on Cropnosis Limited and USDA/NASS. Others include nematicides, fumigants, other miscellaneous conventional pesticides, and chemicals used as pesticides such as sulfur, petroleum oil and sulfuric acid. Wood preservatives, specialty biocides, and chlorine/hypochlorites are not included. (B) Data for cultivated GMOs come from ISAAA global status of commercialized biotech/GM crops. The herbicide tolerance is usually to Roundup, and the modified insecticides are usually from mutated Bt genes.

Rat pellets are mostly constituted of cereals (wheat, maize or barley) and other legumes (such as soybean). These are sprayed with different pesticides according to the methods of cultivation, but also according to the year or location, resulting in different contaminants [6,7]. Pesticides are formulated toxics (Fig 1A), supposed to be specific for plants (herbicides), insects (insecticides) or fungi (fungicides). However, non-target effects of their residues are increasingly being identified at chronic dietary levels [8,9]. Some pesticides are strongly associated with agricultural GMOs, such as Roundup, a glyphosate-based formulation, or mutated Bt toxins (Fig 1B). These GMOs are essentially modified to tolerate and/or produce pesticide residues [10]; they are generally not labelled nor monitored in their countries of production, in particular North and South America for GM soybean or maize. Their general use in rodent diet is not documented. Known dietary toxicants such as heavy metals [11] and dioxins [12] are also important to measure, because these are ubiquitous contaminants.

We collected rodent feed samples from 5 continents, because agricultural practices in various locations may generate different contaminants. Although several batches of the same diets may not be exactly equal in contaminations [15, 16], the multiple sampling performed in this study allows approaching the variability and omnipresence of pollutants in rat diets. We included both rat feed used in regulatory toxicity trials (such as Purina 5002) and in university laboratories, but also by breeding companies, to raise and reproduce laboratory rodents (such as Mucedola TD.2016). In the latter case, rodents are exposed during their whole life cycle and across generations. To estimate the hazards due to chronic exposures to these contaminants in diets, we calculated the chronic non cancer hazard indexes (to take into account general toxicological effects) for all the pollutants measured forming chemical mixtures, as recommended by the United States Environmental Protection Agency (US EPA) [17] and the European Food Safety Authority (EFSA) [18]. It has been used recently to calculate the fish consumer risk for PCBs [19] and the vegetable consumer risk for heavy metals [20]. This approach assumes that simultaneous subthreshold exposures to several chemicals could result in adverse health effects [17].

## Material and Methods

### Rodent feed

Rodent diets were obtained directly from the laboratory using the feed or from suppliers from 5 continents. Tested diets were sampled from North America (Teklad Diets 7913 NIH 31, Wisconsin, USA and Purina 5002 LabDiet, Indiana, USA); Latin America (Purolab 22P, PuroTrato, and NUVILAB CR1, Nuvilab Sogorb Industria e Comércio Ltda., Brazil); Europe (A04 Safe, France; Mucedola S4RF21 and Harlan TD.2016, Italy, as well as Ssniff S8106-S011 and V1326-000, Germany, and 801151 RM1, Special Diet Services, UK); Africa (Belmill Mice pencil, Belfast Millers limited, Kenya); Asia (HFK 1022 Bioscience Co, Beijing, China); and Oceania (Reliance StockFoods R94, New Zealand). Rodent pellets were stored at -80°C upon reception. The sampling in triplicate was precisely performed according to 2002/63/CE guidelines. The country producing the feed may not reflect the place where the cereals were grown since the origins of the ingredients are variable for the suppliers.

### Contaminant analyses

To insure the accuracy and reproducibility of the data (in particular adequate replications, standard deviations and coefficients of variations), all measurements were performed in laboratories accredited by COFRAC, the French accreditation body. Details are given below. The list of contaminants measured is given in Table 1.

**Table 1:** Rodent feed contaminants measured in this study. For techniques, see materials and methods. All measurements were performed in accredited laboratories.

**Pesticides**	*Analyzed by GC/MS*: acrinathrin, aldrin, bifenthrin, bromophos ethyl and methyl, bromopropylate, CHB 26, CHB 50, CHB 62, chinomethionat, chlordane, chlorfenapyr, chlorfenson, chlormephos, chlorobenzilate, chloroneb, chlozolinate, cyfluthrin, cypermethrin, DDD (o,p’ and p, p’), DDE (o,p’ and p, p’), DDT (o,p’ and p, p’), deltamethrin, dichlobenil, dicloran, dicofol, dieldrin, endosulfan (sulphate, alpha and beta), endrin, etridiazole, fenchlorphos, fenitrothion, fenson, fenpropathrin, fenvalerate, fipronil, flucythrinate, HCH (alpha, beta, delta), lindane, heptachlor epoxide (endo and exo), hexachlorobenzene, iprodione, isodrin, isoprothiolane, lambda-cyhalothrin, methoxychlor, nitrofen, nonachlor (cis and trans), parathion (methyl and ethyl), pendimethaline, pentachlorobenzene, permethrin, phenothrin, phorate, procymidone, profluralin, quintozene, resmethrin, tau-fluvalinate, tecnazene, tetradifon, tetramethrin, toclofos methyl, trifluralin, vinclozolin.
	*Analyzed by LC/MS-MS*: acephate, acetamiprid, aclonifen, alachlor, aldicarb, aldicarb sulfone, amitraz, AMPA, atrazine, azinphos (ethyl and methyl), azoxystrobin, benalaxyl, bendiocarb, bifenox, bitertanol, boscalid, bromacil, bromuconazole, bupirimate, buprofezin, carbaryl, carbendazim, carbofuran, carbophenothion, carboxin, chlorfenvinphos, chloridazon, chlorimuron ethyl, chlorpyriphos (methyl and ethyl), chlorthiophos, cinosulfuron, clodinafop-propargyl, clothianidin, coumaphos, cyanazine, cyanofenphos, cyazofamid, cycloxydim, cymoxanil, cyproconazole, cyprodinil, demeton-S-methyl (and sulfone), diallate, diazinon, dichlofenthion, dichlorvos, diclofop methyl, dicrotophos, diethofencarb, difenoconazole, diflufenican, dimethachlor, dimethoate, dimethomorphe, dioxathion, disulfoton, ditalimphos, EPN, epoxiconazole, ethiofencarb, ethion, ethofumesate, ethoprophos, etofenprox, etrimfos, famoxadone, fenamiphos, fenarimol, fenazaquin, fenbuconazole, fenhexamid, fenoxycarb, fenpropidin, fenpropimorph, fenpyroximate, fenthion (sulfone and sulfoxyde), flufenacet, flufenoxuron, fluquinconazole, flurtamone, flusilazole, fomesafen, fonofos, glyphosate, heptenophos, hexaconazole, hexazinone, hexythiazox, imazalil, imazosulfuron, imidacloprid, indoxacarb, iprovalicarb, isofenphos, isoproturon, kresoxim-methyl, linuron, lufenuron, malaoxon, malathion, mecarbam, mepanipyrim, metalaxyl, metamitron, metazachlor, methabenzthiazuron, methamidophos, methidathion, methiocarb, methomyl, methoxyfenozide, metobromuron, metolachlor, metribuzin, metsulfuron-methyl, mevinphos, monocrotophos, myclobutanil, nuarimol, omethoate, oxadixyl, oxamyl, paclobutrazol, paraoxon (ethyl and methyl), penconazole, phenthoate, phosalone, phosmet, phosphamidon, picoxystrobin, piperonyl butoxide, pirimicarb, pirimiphos-ethyl and methyl, prochloraz, profenofos, promecarb, prometryn, propamocarb, propargite, propazine, propiconazole, propoxur, propyzamide, prosulfuron, prothiofos, pymetrozine, prosulfuron, prothiofos, pymetrozine, pyraclostrobin, pyrazophos, pyridaben, pyridaphenthion, pyrifenox, pyrimethanil, pyriproxyfen, quinalphos, quinoxyfen, simazine, spinosad, spiroxamine, sulfosulfuron, sulfotep, tebuconazole, tebufenozide, tebufenpyrad, terbacil, terbufos, terbuthylazine, terbutryn, tetrachlorvinphos, tetraconazole, thiabendazole, thiacloprid, thiamethoxam, thifensulfuron-methyl, thiofanox, thiometon, triadimefon, triadimenol
**GMO events**	Soy : RRS1, RRS2 ; maize : Cry1a, MON810, MON863, MON 88017, MON 89034, T25, TC1507, Bt11, DAS 59122, GA 21, MIR 604, MIR162, NK 603 ; oilseed rape : GT73, T45, MS8RF3 ; sugar beet : H7-1 ; potato : H92-527-1 ; rice : Bt63 ; wheat : MON71800
**Heavy metals**	lead (Pb), cadmium (Cd), mercury (Hg) and arsenic (As)
**PCDD/Fs, PCBs**	*PCDD/Fs*: 2,3,7,8-TCDF; 2,3,7,8-TCDD; 1,2,3,7,8-PeCDF; 2,3,4,7,8-PeCDF; 1,2,3,7,8-PeCDD; 1,2,3,4,7,8-HxCDF; 1,2,3,6,7,8-HxCDF; 2,3,4,6,7,8-HxCDF; 1,2,3,7,8,9-HxCDF; 1,2,3,4,7,8-HxCDD; 1,2,3,6,7,8-HxCDD; 1,2,3,7,8,9-HxCDD; 1,2,3,4,6,7,8-HpCDF; 1,2,3,4,7,8,9-HpCDF; 1,2,3,4,6,7,8-HpCDD; OCDF; OCDD *PCBs dioxin-like*: PCB 81; PCB 77; PCB 126; PCB 169; PCB 123; PCB 118; PCB 114; PCB 105; PCB 167; PCB 156; PCB 157; PCB 189; *PCBs indicators*: PCB 28; PCB 118; PCB 52; PCB 101; PCB 138; PCB 153; PCB 180

#### Pesticides residues measurements

100 g of each sample were grinded in a MaxiGrinder Solo (Genomic Industry, Archamps, France) to ensure homogeneity and representativity, and 5 g of this homogenate were extracted. Residues of 262 pesticides (see Table 1 for the detailed list) were measured once by sample by a multi-residue GC-MS and/or LC-MS/MS method following acetonitrile extraction/partitioning and clean-up by dispersive solid-phase extraction—QuEChERS-method [21] (European and French Standard NF EN 15662 from January 2009 for foods of plant origin). Limits of quantifications (LOQ) varied from 10 to 100 ppb according to each pesticide; limits of detections (LOD) were the third of LOQ. Glyphosate (G) and its degradation product aminomethyl phosphonic acid (AMPA) were determined by isotope dilution and solid-phase extraction and LC-MS/MS. They were extracted with water after addition of internal standards of stable C^13^-isotopes. Aliquotes were derivatized using 9-fluorenylmethyl chloroformate (FMOC), then purified and concentrated on solid-phase extraction cartridges. After filtration, the extracts were injected in LC-MS/MS with electrospray ionization in negative-ion mode using multiple reaction monitoring. Analyses were performed “one-shot”. LOD and LOQ for the sum glyphosate + AMPA were respectively 25 and 50 ppb. Fidelity criteria had been defined previously, during validation. Uncertainties of measurement (including SDs) were calculated from the Horwitz equation [22], they ranged from 16 to 32%, which remains one of the most used at a regulatory level, even if debated [23].

#### GMO quantifications

GMO quantifications were performed by qPCR according to norms ISO 21569, 21570, 21571 and 24276. 100g of samples were grinded and homogenized in a GM 200 grinder (Retsch, Germany). 200 mg were used to extract DNA with QIAsymphony DNA mini kit (Qiagen, Germany) and each subsample was amplified twice by real-time PCR Rotor Gene (Qiagen, Germany). LOD and LOQ were respectively 0.01 and 0.1% (determined on IRMM or AOCS standard material). The global uncertainty of measurements was calculated by adding uncertainty of measurements of the methods (calculated with the standard deviation obtained on certified materials) and standard deviation of the 4 repeats per sample. These global uncertainties ranged from 20 to 33%. Plant contents were first determined by a screening PCR for taxon-specific markers (HMG for maize, lectin for soy, acc for oilseed rape, gluA3 for sugar beet, Qgene for wheat, acp1 for cotton, UGPase for potato). Then the presence of GM material was assessed by a screening PCR for generic recombinant markers (CAMVp35S, Tnos, FMVp35S) and 22 GM-specific events (GMOs authorized in diets imported in European Union) were searched in maize (12) and soy (2) for all the diets, and in addition oilseed rape (3), potato (1), sugar beet (1) and non-commercialized GMOs like rice and wheat, in the 3 diets containing the highest proportion of GMOs.

#### Heavy metals measurements

100 g of each sample were grinded in a MaxiGrinder Solo (Genomic Industry, Archamps, France). The content of arsenic, cadmium, lead were determined in duplicate using 1g of the homogenate by graphite furnace atomic absorption spectrometry according to norm NF EN 15550 (November 2007). Mineralization is performed using 48-position DigiPREP (SCP Science, Courtaboeuf, France) under atmospheric pressure, then the content in arsenic, cadmium and lead of the mineral deposit is measured by graphite furnace atomic absorption spectrometry (GF-AAS) or AAFG Zeeman for arsenic. Wavelengths are 193.7 nm for arsenic, 228.8 nm for cadmium and 283.3 nm for lead. Limits of quantification were respectively 100, 10, 100 ppb. The global uncertainties (including SDs) were 33% for arsenic, between 10 and 50% for cadmium and between 38 to 46% for lead.

Mercury is quantified according to a method derived from the Method US EPA 7473. Diet samples are dried, then submitted to thermal decomposition in order to release the mercury vapours and amalgamate them on threads of gold. Then the threads of gold are thermally desorbed and the mercury released quantified by GF-AAS. Wavelength is 253.65 nm and limit of quantification is 5 ppb. The global uncertainties were equal to 20%.

#### PCDD/Fs and PCBs measurements

The determination of PCDDs, PCDFs and PCBs in animal feed is done by using the GC/HRMS technique in combination with the isotope dilution. The sample preparation and analysis is based on the EPA method 8290. ^13^C-labelled 2,3,7,8-chlorinated PCDD and PCDF congeners and ^13^C-labelled PCB congeners are added in different stadia of the sample preparation in order to correct for possible losses. First, the animal feed sample is grinded using a Retch grinding system. The grinded sample is extracted by soxhlet for 20 hours, after the ^13^C-labelled extraction standard solution was added. The fat, present in the evaporated extract of the soxhlet solution, is destructed with acid silica followed by a mixed acid and basic silica clean up and an alumina clean up. The becoming extract is evaporated and injection standard is added just before injection on the GC/HRMS system.

Second, matrix interferences are removed by a clean-up using a multilayer column and alumina DB5MS column. Finally, the concentrated extract is injected on GC/HRMS (GC 6890 from Agilent and MS Autospec Ultima series from Micromass). The chromatographic process separates the specifically sought congeners from the others. Mass spectrometric parameters gives a separation between the PCDD’s, PCDF’s and PCB’s, between the different chlorination degrees and between the ^13^C-labelled and ^12^C-native congeners by using selected ion recording at resolution 10 000 of two selected ions of each congener. The content of PCDD/Fs and PCBs were determined HRGC/HRMS. The limit of quantification (LOQ) was 0.05 pg/g or ppt (except OCDD/F = 0.1 ppt) for individual congeners involved in the dioxins/furans (PCDD/Fs) content. For dioxin-like PCBs, the LOQ was 0.05 ppt for non-ortho PCBs and 10 ppt for mono-ortho PCBs. The LOQ was 100 ppt for indicator PCBs. The global uncertainties (including SDs, calculated from the Horwitz equation) were around 30% in all cases.

The most important quality control checks are 1/ the separation between 1,2,3,4,7,8-HxCDD and 1,2,3,6,7,8-HxCDD and between PCB-123 and PCB-118; 2/ the deviation of the native and labeled PCDD’s, PCDDF’s and PCB’s of the calibration control is checked against the calibration curve; 3/ the isotopic ratios between the different ions do not differ more than 20% compared to the theoretical ratios; 4/ the retention time of the native congeners is checked against the retention time of the labeled congeners; 5/ the recovery of the extraction standard is controlled; 6/ control samples are analyzed and similar to a control chart; 7/ drift control is checked.

### Calculations of Hazard Quotients, Indexes, and statistical analyses

We calculated the hazard quotient (HQ), as the ratio of the potential chronic daily intake of each substance to the corresponding chronic reference dose at which no adverse effect is supposed to be expected, such as the Acceptable Daily Intake (ADI). The hazard index (HI) is the sum of HQ. This method is recommended by US EPA [17]. Thus, the chronic daily intake is first deduced from the conversion factor 0.05 calculated from 37 chronic studies (used also for ADI determination in rats) by EFSA [24]. For instance, a concentration of 1 ppm of contaminant in feed is equivalent to a dose of 0.05 ppm body weight / day for adult rats. We calculated the sums of HQ (ΣHQ) for all measured known toxicants. However, these correspond to an underestimation of chronic toxic effects, since all toxicants cannot be known. As an example, adjuvants giving a non-additive but multiplying effect in some cases [25] are not taken into account. The correlation coefficient between Roundup-tolerant GMOs and some Roundup residues (G + AMPA) was calculated according to Pearson product—moment correlation coefficient using Stata/IC 12.1 [26].

## Results

### Pesticides

**Fig 2:**
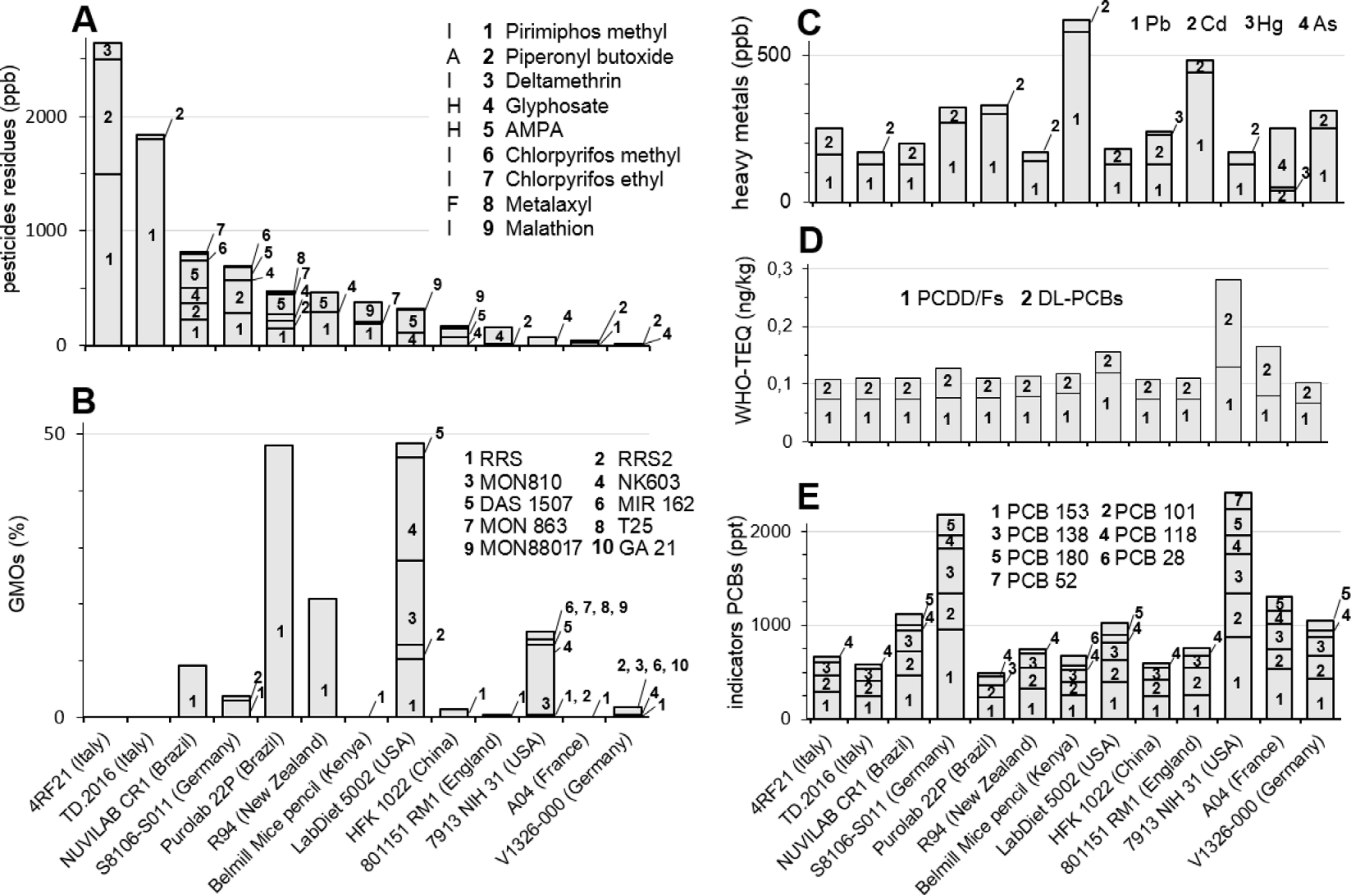
Environmental contaminants in 13 rodent diets used worldwide. Countries indicate the feed manufacturing locations, which can differ from the cultivations. (A) 262 pesticides have been measured in ppb (μg/kg) by multi-residue GC-MS and/or LC-MS/MS. F Fungicide, H Herbicide, I Insecticide. (B) GMOs quantifications (for 23 events) were performed by qPCR. Confidence intervals for GMOs were in average 35% per assay. (C) Heavy metals contents (ppb or μg/kg) were determined by Graphite furnace atomic absorption spectrometry. (D) PCDD/Fs + DL-PCBs are measured in ng TEQ/kg wet weight. (E) Indicators PCBs are measured in ppt (ng/kg dry weight). Reproducibility relative standard deviation are not indicated to improve readability, they have been calculated from fidelity data on repeatability and reproducibility within the laboratory (around 40% per assay for heavy metals, 20–30% for pesticides, 27–31% for PCDD/fs + DL-PCBs and 30 to 35.8% for indicators PCBs).

First of all, it appears that all the samples were contaminated by pesticides residues (Fig 2A), the 2 samples from Italy being the most contaminated (up to 2641 ppb). The contamination was very heterogeneous, with 1–6 different residues per feed, the highest number of different pesticide residues being detected in NUVILAB CR1 (Brazil). Pirimiphos methyl was the most frequent residue detected in 8 out 13 feeds, at levels up to 1800 ppb in TD.2016 (Italy). 7 diets exceeded the ADI (Table 2), thus the HQ was above 1, up to 22.5 in TD.2016 (Table 3). Out of the 9 pesticide residues detected, 5 were insecticides (pirimiphos methyl, deltamethrin, chlorpyrifos methyl and ethyl, and malathion), 2 came from the herbicide Roundup or of other glyphosate-based herbicides (glyphosate and its metabolite AMPA). There was only one fungicide residue, metalaxyl. Piperonyl butoxide, detected in 8 samples, is used as a synergist in various pesticides.

**Table 2:** Maximum residue levels and daily intakes of contaminants in 13 rodent diets used worldwide in comparison to regulatory limits. N: Number of diets in which a contaminant was detected. Max: Maximal quantities measured in the diets (diets over MRL in parentheses). MRL: Maximum Residue Levels. Max DI: calculated maximal daily intake (diets over ADI in parentheses). ADI: Acceptable Daily Intakes. NE: not existing. For pesticides and heavy metals, residue levels are in μg/kg (ppb) with MRLs for various cereals (EC/1107/2009, EU Pesticides database (available at www.ec.europa.eu/sanco_pesticides/public/) and Codex Standard 193–1995 respectively; ADIs are in μg/kg/bw/d (EFSA or FAO/WHO). For dioxins (PCDD/Fs) and DL-PCBs, residue levels are in ng TEQ /kg wet weight, indicators PCB (PCBi) are in ng/kg wet weight. The corresponding MRLs are for feed of plant origin with a moisture content of 12% (EU 277/2012). ADIs are in pg TEQ/kg/bw/d for PCDD/Fs and PCDD/Fs + DL-PCBs (EC 1881/2006) and in ng/kg/bw/d for the sum of the 6 PCBi (Afssa 2006-SA-0305, http://www.anses.fr/Documents/RCCP2006sa0305b.pdf).

		N	Max	MRL	Max DI	ADI
**Pesticides**	Pirimiphos methyl	8	1,800	5,000	**90 (7)**	4
	Piperonyl butoxide	8	1,000	NE	50	200
	Deltamethrin	1	141	2,000	7	10
	Glyphosate + AMPA	9	370	100–20,000	18,5	300
	Chlorpyrifos methyl	2	59	3,000	3	10
	Chlorpyrifos ethyl	3	23	3,000	1.5	10
	Metalaxyl	1	20	50	1	80
	Malathion	3	170	8,000	8.5	30
**Metals**	Pb	12	**580 (5)**	200	**29 (12)**	3.57
	Cd	13	**100 (1)**	100	**5 (13)**	0.357
	Hg	2	10	100	0.5	0.571
	As	1	**200 (1)**	100	**10 (1)**	2.14
	**PCDD/Fs**	13	0.13	0.75	**6.5 (13)**	1
	**PCDD/Fs+DL-PCBs**	13	0.28	1.25	**14 (13)**	2
	**Σ_6_ PCBi**	13	1950	10000	**97.5 (13)**	10

**Table 3:** Hazard quotients (HQ) and ΣHQ for 13 rodent diets used worldwide. HQ were calculated for each contaminant according to regulatory guidelines for chronic non-cancer risk characterization (EPA, Risk Assessment Guidance for Superfund RAGS Part A, Chapter 8, http://www.epa.gov/oswer/riskassessment/ragsa/). HQ is the ratio of the chronic daily exposure and the ADI of each contaminant. The sum of calculated HQ (ΣHQ) is indicated.

	Pesticides							Metals				Dioxins+PCBs		ΣHQ
Hazard Quotient (HQ)	Pirimiphos methyl	Piperonyl butoxide	Deltamethrin	Glyphosate+ AMPA	Chlorpyriphos	Metalaxyl	Malathion	Pb	Cd	Hg	As	ΣPCDD/Fs + DL-PCBs	Σ_6_PCBi	
4RF21 (Italy)	18.8	0.3	0.7	0	0	0	0	2.2	12.6	0	0	2.7	3.3	40.5
TD.2016 (Italy)	22.5	0	0	0	0	0	0	1.8	5.6	0	0	2.8	3.0	35.6
NUVILAB CR1 (Brazil)	2.9	0	0	0.1	0.4	0	0	1.8	9.8	0	0	2.8	5.1	22.8
S8106-S011 (Germany)	3.5	0.1	0	>0	0.1	0	0	3.8	7.0	0	0	3.2	9.5	27.1
Purolab 22P (Brazil)	1.9	0	0	0	0.1	0	0	4.2	4.2	0	0	2.8	2.7	15.8
R94 (New Zealand)	3.6	0	0	>0	0	0	0	2.0	4.2	0	0	2.9	3.7	16.3
Belmill Mice pencil (Kenya)	2.4	0	0	0	0.1	0	0.3	8.1	5.6	0	0	3.0	3.2	22.6
LabDiet 5002 (USA)	0	0	0	>0	0	0	0	1.8	7.0	0	0	3.9	4.5	17.3
HFK 1022 (China)	0	0	0	0	0	0	0	1.8	14	0.9	0	2.7	3.0	22.5
801151 RM1 (England)	0	0	0	0	0	0	0	6.2	5.6	0	0	2.8	3.6	18.1
7913 NIH 31 (USA)	0	0	0	0	0	0	0	1.8	5.6	0	0	7.0	9.8	24.2
A04 (France)	0.3	0	0	0	0	0	0	0	5.6	0.9	4.7	4.2	5.5	21.1
V1326-000 (Germany)	0	0	0	>0	0	0	0	3.5	8.4	0	0	2.6	4.7	19.1

### GMOs

This is the first study reporting the extent of the worldwide use of GMOs in rodent diet (Fig 2B): 11/13 samples were positive except the 2 Italian diets. Labelling is compulsory only in the European Union above 0.9% per ingredient. On 4 European GMO-containing diets, 2 German diets were labelled as such. The French A04 diet had only 0.3% GM soy, but the English 801151 RM1 diet had 32 ± 8% GM soy (0.47% of the total) and was not labelled. The Brazilian Purolab 22 P was labelled (48% GM soy) but the US Purina 5002 LabDiet had around 12.8% GM soy and 35.6% GM maize and was not labelled. In this analysis, all GM events were measured, whether stacked or not. Out of 22 specific GM events searched for, 12 were detected: 8 GM maize, 2 GM soy and 2 GM oilseed rape varieties. Among those GM events, 6 were Roundup tolerant (RRS1 and RRS2 soy, GA21, MON88017 and NK603 maize, and GT73 oilseed rape), 3 were glufosinate tolerant (DAS1507 and T25 maize, MS8RF3 oilseed rape), and 5 produced modified Bt toxin (DAS1507, MIR162, MON810, MON863, and MON88017 maize). 2 have stacked events (DAS 1507 and MON88017). Except for a tiny amount of GM oilseed rape (0.07–0.7%), it appears that the majority of GMO content is soya or maize, but their amount is highly variable (0–48%). North and South American as well as New Zealand feeds have the highest GMO content. 48% of the Purolab 22P (Brazil) diet is composed of Roundup Ready soybeans. The same global content of GMOs is in the LabDiet 5002 (USA) but is composed of 7 events (2 traits for soy, 3 for maize, 2 for oilseed rape). 9 different GM events were maximally detected in the 7913 NIH 31 feed (USA), including in total at least 15% GMOs, composed of RRS1 and RRS2 (soy), and MON810, MON863, NK603, T25, Mir162, MON88017 and DAS 59122 (all these 7 events are in various GM maize varieties). GMOs were detected less frequently or not at all in European feeds, and were absent from the African feed (GMOs cultivated in Africa mostly consist of GM cotton).

**Fig 3:**
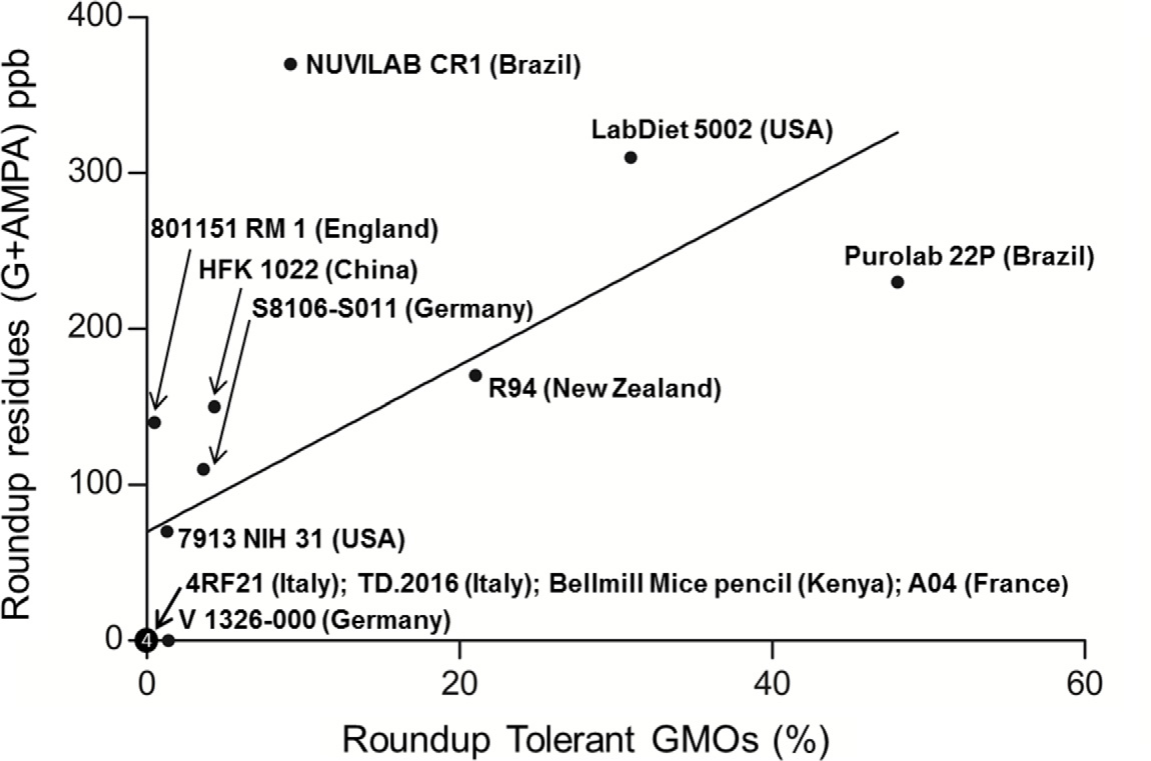
Roundup residues (glyphosate + AMPA, ppb or μg/kg) as a function of Roundup-tolerant GMOs quantities (%) in 13 rodent diets used worldwide. The linear regression was calculated in Stata (y = 5.34x + 69.97), the Pearson’s r indicates a significant correlation (r = 0.64, p = 0.019). The y-axis is labelled as such because while other glyphosate-based herbicides do exist, legally only Roundup should be used on glyphosate-tolerant plants due to commercial agreements. However, other glyphosate-based herbicides may be used in some countries.

Agricultural GMOs are mostly tolerant to Roundup. The maximum glyphosate herbicide residues detected were 130 ppb of glyphosate and 240 ppb of AMPA in NUVILAB CR1 (Brazil). The second highest herbicide content (310 ppb) was in the LabDiet 5002 (USA). As a matter of fact, glyphosate and AMPA, the only herbicide residues detected, were only found in Roundup-tolerant GMO-containing diets, and no herbicides were detected in other samples. There is even a positive correlation (Pearson’s r = 0.64, p = 0.019) in all diets between the content of all Roundup tolerant GMOs and R residues (G+AMPA), as shown in Fig 3.

### Heavy metals

In contrast with pesticides and GMOs, contaminations by heavy metals (Fig 2C) appear more homogenous. The content of lead is the most variable (up to 580 ppb for Kenyan and 440 ppb in UK diets), in contrast with cadmium (30 to 100 ppb). The Chinese and the French feeds were contaminated with low levels of mercury (10 ppb), the French feed being also contaminated with arsenic (200 ppb). Regarding only heavy metals, the ΣHQ vary from 6.2 to 16.7 (Table 3).

### Dioxins and PCBs

Dioxins and PCBs were detected in all samples (Fig 2D and 2E). The contents in PCDD/Fs range between 0.067 ± 0.019 ng TEQ/kg wet weight (12% moisture) (V1326-000, Germany) and 0.130 ± 0.039 (7913 NIH 31, USA). In these diets, the sum of PCDD/Fs and DL-PCBs are 0.102 ± 0.029 and 0.280 ± 0.084 respectively, representing the minimum and maximum for all diets. The sum of the 6 indicators NDL-PCBs (PCB-118 excluded) shows even more variable contents in diets: from 530 ± 310 ng/kg wet weight (PuroLab 22P, Brazil) up to 1,950 ± 640 (again in the diet 7913 NIH 31, USA). The chronic consumption by rats of all of the diets exceeds the ADI for PCDD/Fs, and for the sum PCDD/Fs + DL-PCBs, and also for NDL-PCBs (Table 2). Only for these contaminants, the ΣHQ vary from 5.4 to 16.8 (Table 3).

### ΣHQ for all contaminants

All diets consumed chronically reach very high ΣHQ (15.8–40.5; the risk has to be carefully considered above 0.2 or 1).

## Discussion

This is the first report of the global precisely measured contamination of laboratory rodent diets throughout the world by pesticides, GMOs, heavy metals, dioxins and PCBs. Health effects of most of these products including at the levels we found are documented, even if usually the mixtures effects under the official thresholds [27,28] are not assessed. This is why we calculated here HQ and their sums. The results of this work are in agreement with the CONTAM panel of EFSA [29], which concludes that animal health risk assessment is still accompanied by a high degree of uncertainty and needs further development.

The contaminants and their levels found in this work are consistent with various other studies. For instance, Zeljenková et al. [30] measured in lab rodent diet for a 90 day GMO study 530–1600 ppb of pirimiphos-methyl, 94–300 ppb of piperonyl butoxide, 118–173 ppb of arsenic, 0.16 ng TEQ dioxins /kg, like in Schecter et al. [31] and in this work.

The diet 4RF21 (Italy), which is the most contaminated by pesticides, contains 1500 ppb of pirimiphos-methyl; this corresponds to a chronic daily intake by rats of 75 ppb/bw/d, according to default calculated values [24]. This pesticide is known to inhibit plasma cholinesterase in rats treated at 200 ppb/bw/d [32]. Other effects could happen at lower doses, though these have not been tested, since toxic effects may arise in non-linear manner [33]. However, this intake is already 19-fold above the ADI. In addition, this diet is also contaminated with other pesticides such as deltamethrin (141 ppb), which corresponds to a chronic daily intake by rats of 7 ppb/bw/d. Deltamethrin is a tumor initiator in Swiss albino mice from 4 ppm/bw/d [34]. It is characterized as a neurodevelopmental toxicant in rats from 80 ppb/bw/d [35]. In fact, the risk is also amplified by mixture effects because the same diet contained 1 ppm (50 ppb/bw/d in rats) of the synergist piperonyl butoxide, which is added to pesticide formulations to increase their toxicities [36], in particular in pyrethrinoids such as deltamethrin. Adjuvants are developed to amplify the toxic effects on plants, insects, or fungi, and also amplify toxic effects in mammals [25,37]. For insecticides alone together with the piperonyl butoxide, the ΣHQ (sum of ratios of each exposure to corresponding ADI) of this diet is already 19.7, and for all contaminants taken together it reaches 40.5 (Table 3), which is at least 40 times over the limit of concern. In addition, this does not take into account the potential synergistic effects.

A 100 safety factor (under the no observed adverse effect level) is generally applied for the ADI calculation. It takes into account both intra (x10) and interspecies (x10) variability. Since we are in all cases above 10 in Table 3 (15.8–40.5), these diets may represent a risk for some species or for some animals within the species, and moreover we did not measure all other possible contaminants. All these considerations could explain, by themselves, a possible rate of chronic pathologies reported in rodents eating these diets. Given that combinations of contaminants are likely to change over time and location, the rates of chronic pathologies would not be stable in control rats across different experiments. As a consequence, historical control data are unsuitable to be used as general controls.

The presence of piperonyl butoxide, a pesticide synergistic compound, in 8 of the 13 feeds tested, implies that pesticides should be tested in formulation and not as single compounds. Adjuvants are rarely monitored, but some widely used adjuvants (surfactants) such as nonylphenol ethoxylates, are widely found in the environment and are linked with wildlife endocrine and reproductive disruptions [38]. Among other pesticides measured, chlorpyriphos-methyl is an endocrine disruptor and induces anti-androgenic effects and hypothyroidism from prenatal exposure [39]. The presence of these residues could also explain the high levels of mammary or pituitary tumors in rat control populations [40]. Pesticides like malathion, or chlorpyrifos for instance, induce changes in the rat mammary gland [41,42]. Glyphosate induces human breast cancer cells growth through estrogenic pathways at levels as low as 0.1 ppb [43], as does Roundup at a comparable level for mammary adenomas growth in vivo [44]. Glyphosate is described as a tumor promoter [45]. Even if more sampling are desirable, the correlation between the quantity of Roundup residues and Roundup-tolerant GMOs (the majority of GMOs cultivated, namely NK603, RRS1, and RRS2) indicates that glyphosate residues are found in agricultural cultivations of Roundup-tolerant GMOs, mostly soy and maize. This was already described by Bohn et al. [46]. Only the Brazilian diet NUVILAB CR1 has double or triple levels of residues than those expected with the linear regression; this could suggest a higher number of sprays or a greater quantity of herbicide used in the field, and/or contamination of water or soil due to its persistence, as already documented [47].

There is no other correlation between GMO content and total pesticide residues (Pearson’s r = -0.21, p = 0.49). The 4 diets with the highest GM content (9–48%) come from America and New Zealand; this is logical because the American continent produces 95% of edible GMOs, excluding cotton [10]. 80% of them are Roundup tolerant, it was thus not surprising that glyphosate and its metabolite AMPA coming for Roundup were detected in the North and South American rodent feed, where the majority of cultivations of these crops are GM. Even if GMO toxicities remain controversial [48], the contamination of 9/13 samples is an issue of concern because some feed like the Purina 5002, containing 48% GMOs, is used regularly as a control in toxicological tests of GMOs [49]. In this last case, no data on the presence of Roundup-tolerant GMOs or on Roundup residues in the feed was provided although they are critical information for safety conclusions [50]. Generally, in these tests, only the GM plant is characterized for GMO content, while the quantity of GMOs in the rest of the feed remains unknown.

Concerning the general contamination of all the diets by bioaccumulative heavy metals, it is astonishing that consumption of 12 out of the 13 diets exceeded the ADI for Pb, and all for Cd, with an important exceedance of the standard HQ (3.0 ± 2.2 and 7.3 ± 3.1, respectively). Even if rat diets do not follow the human standards, 7 overpassed the human MRL for lead content in various cereals (2 from Germany, and 1 from Kenya, UK, and Brazil), or for arsenic (France) or cadmium (China) contents (Table 2). The daily intake by rats of all these diets exceeds the human ADI for cadmium, and for lead this is the case for 12 diets out of 13. This may by itself explain at least in part some cancers or other diseases, including mammary tumors in animal controls using these diets, especially because Cd is an estrogen-like compound [51]. The potential impact of dietary differences in nutritional components and contaminants, including pesticides and heavy metals, has been known to be an issue for some time [52,53]. It has already been demonstrated that arsenic contamination of control diets confounds risk assessment for low dose effects of heavy metals [54]. The French rodent diet had levels of arsenic (200 ppb) in the range of this former study, in which mice fed an arsenic-contaminated diet presented an increase (up to 37-fold) in the gene expression of genes involved in xenobiotic (cytochromes P450) and glutathione metabolism.

Similarly, consumption of all diets exceed the ADI for dioxins, furans and dioxin-like PCBs (PCDD/Fs and DL-PCBs) and also for NDL-PCBs used as indicators (PCBi), with the HQ for these 2 groups varying from 2.6 to 7.0 (mean 3.3 ± 1.2) and from 2.7 to 9.8 (mean 4.7 ± 2.3), respectively. Their health effects are increasingly documented [55], in particular as hepatotoxics and endocrine disruptors as well as immunosuppressors at very low levels in the range of the contaminations evidenced in our study or in a rat diet tested by Schecter et al. [56]. These products are known to bioaccumulate during chronic exposure [55]. They may certainly contribute to pathologies in the rats fed with these diets.

Laboratory rodents are also contaminated with plasticizers released by cages or from water sources [57–58]. In one case the endocrine disrupting effect of plasticizers was discovered because of disturbed endocrine function in control groups [59].

It becomes clear that even if the background of pathologies of control laboratory rodents is due, at least in part, to *ad libitum* feeding or inactivity in closed cages, inducing obesity, as previously suggested [60], the contamination of their diet cannot be excluded as a cause. Some pollutants are even obesogens by themselves [61]. Almost all laboratory rats are inactive and fed *ad libitum*, thus these factors alone cannot explain the huge differences within controls in toxicological historical records [3]. For instance, the incidence of mammary fibroadenomas among populations of Charles River Sprague-Dawley females ranged from 13 to 62% [3]. In fact, it is already known that the same feed ingredients produced with different cultivation methods differently impaired biochemical markers of rats’ health [62]. Epigenetic consequences are also documented in numerous cases for all these classes of pollutants, inducing possibly transgenerational effects in the rat strains [63–65].

Taken together, these data may challenge the use of external control groups in regulatory chronic health risk assessments, because differential diet contaminations artificially enhance background effects and hide significant effects. It is thus inappropriate to combine different controls from different experiments within the same laboratory because different batches of the same feed may not be always similarly contaminated over time. Differences in feed constituents between countries may be even more important. This background rate of pathologies involves the use of huge numbers of animals to detect statistical differences in tumor incidence in chronic toxicity tests. The organisation for economic co-operation and development 453 guideline on carcinogenicity test stipulate the use of at least 50 animals per group to detect carcinogenic effects. This is because the statistical power needed to detect a significant difference between treated and controls is lost due to the high level of tumors in controls. Thus an increase of the number of animals per group is proposed [66]. However, this is not coherent with current concern about animal welfare. Even if basic tumorigram of the various species and strains would results in different rates of tumors, it would be better to minimize the background rate of pathologies by using a safe, non-polluted diet, and to restrict comparisons only to the concurrent matched controls of an experiment.

Even if these results throw into question the value of animal feeding trials performed to date, we are not in favour of the reduction of toxicological assessment to solely in vitro systems. Embryonic stem cell tests can be a valuable first step in identifying embryonic toxicants, but they do not perfectly reproduce embryotoxicity [67] and will never replace in vivo developmental toxicity testing. Furthermore, for agents to which all people at all ages may be exposed, it should be considered to start the exposure from prenatal life to allow carcinogenic potential to express its effects during the most vulnerable part of the development [68].

In conclusion, the fact that all laboratory rodent diets tested are contaminated by toxic environmental chemicals (mean ΣHQ = 23 ± 7, far above the preoccupying level of 1) has huge consequences to current practices in biomedical research. Moreover, the HQ is enhanced for mouse, young rats or depending on the periods of exposures during development because their daily intake is more important [24]. All these data taken together invalidate the use of historical control data and questions the use of at least 50 rats per group in carcinogenicity studies. Similarly and very recently, Kuroiwa et al. [69] concluded that the diverse and fluctuant incidences of pathologies in historical data may be caused by environmental factors, rather than “spontaneous” or genetic causes in F344 rats. Efforts towards safer agricultural practices and better control of environmental contaminants have to be made in order to feed laboratory rodents with healthy diets. This will not only improve the reliability of toxicity tests, but also the value of animal feeding trials in biomedical research.

## Supporting Information

S1 Data Raw Data. (XLSX)
