# Correction: Laboratory Rodent Diets Contain Toxic Levels of Environmental Contaminants: Implications for Regulatory Tests

**DOI:** 10.1371/journal.pone.0135542

**Published:** 2015-08-07

**Authors:** The PLOS ONE Staff

## Notice of Republication

This article was republished on July 23, 2015, to replace the declaration of Competing interests, which was incorrect. While this article was in production, in-house editorial staff noted that the declaration of competing interests supplied by the authors required revision in order to ensure compliance with the journal’s editorial policies. The editorial team followed up with the authors and the declaration was updated; unfortunately this updated declaration of competing interests was not reflected in the published article.

The republished article includes the correct declaration of Competing interests, as below:

**Competing interests:** The authors have received funding for this and earlier research from CRIIGEN, the Foundation Lea Nature and Malongo, the JMG Foundation and Foundations Charles Léopold Mayer for the Progress of Humankind, Nature Vivante, Denis Guichard, Institute Bio Forschung Austria, and the Sustainable Food Alliance. The laboratory received funding from Sevene Pharma in the last five years to study the detoxifying capacity of plant extracts on Roundup residues, bisphenol A and atrazin. Prof Seralini participated and received payment for a lecture organized by Sevene Pharma.

## Supporting Information

S1 File Originally published, uncorrected article. (PDF)

S2 File Republished, corrected article. (PDF)
